# Comparative analysis of sulfuric acid and free cellulase hydrolysis for waste-paper-to-glucose conversion: experimental and techno-economic evaluation

**DOI:** 10.1098/rsos.241810

**Published:** 2025-06-25

**Authors:** Mahfuzah Samirah Ideris, Mohd Hafez Mohd Isa, Mohd Muzamir Mahat, Jason P. Hallett, S. M. Shahrul Nizan Shikh Zahari

**Affiliations:** ^1^ Industrial Chemistry Technology Programme, Faculty of Science and Technology, Universiti Sains Islam Malaysia, Bandar Baru Nilai, Negeri Sembilan 71800, Malaysia; ^2^ Faculty of Applied Sciences, Universiti Teknologi MARA, Shah Alam, Selangor 40450, Malaysia; ^3^ Department of Chemical Engineering, Imperial College London, South Kensington Campus, London SW7 2AZ, UK

**Keywords:** Hydrolysis, waste paper, glucose, sulfuric acid, free-cellulase enzyme, techno-economic analysis

## Abstract

The approach of converting waste paper (WP) to glucose via hydrolysis reaction is a promising alternative to current disposal methods. Glucose is a key intermediate in the production of various chemicals. In this study, we first characterize WP by several analytical tools and then compare the WP-to-glucose conversion via sulfuric acid (SA)- and free cellulase enzyme (FE)-catalysed reactions, primarily focusing on experimental and techno-economic evaluation. WP contains loosely connected fibres with inorganic filler elements (Ca, Si and Al) distributed on their surfaces. SA-hydrolysis completely hydrolyses WP to glucose in just 2 h; however, the use of H_2_SO_4_ and CaCO_3_, the production of CO_2_ and CaSO_4_ by-products, as well as the complex procedure significantly increase the projected operating costs, leading to substantial profit loss. Similarly, FE-hydrolysis converts all cellulose in WP into 100% glucose, as other components (hemicellulose, lignin and inorganic fillers) appear not to impede cellulose-enzyme binding, while loosely cross-linked fibres further enhance enzyme interaction. With water as the waste, FE-hydrolysis is projected to have much lower operating costs, contributing to *ca* 400 times lower profit loss compared with SA-hydrolysis. However, the 72 h hydrolysis period and the difficulty in separating cellulase enzyme from glucose solution present significant challenges, necessitating further process improvements.

## 1. Introduction

Over the past few decades, there has been a significant increase in the consumption of paper in line with economic growth. It has been reported that the world’s paper consumption reached 417 million metric tons in 2021 and is expected to rise over the next several decades to exceed 476 million metric tons by 2032 [1]. Current waste-paper (WP) disposal technologies, such as incineration and landfilling, have several drawbacks. They include air pollution and soil contamination [2]. Interestingly, WP is made up primarily of cellulose fibres that can be converted to glucose, a high-value intermediate for the production of various chemical products [3,4].

Cellulose consists of linear chains of β-glucose units linked covalently by 1,4-glycosidic (C-O-C) linkages, resulting in chains packed into partially crystalline microfibrils. Within a microfibril, the chains are arranged in sheet, with H-bonding between chains and monomers of each chain [3,5]. Hydrolysing all the 1,4-glycosidic linkages yields glucose. Sulfuric acid (H_2_SO_4_)-catalysed reaction is widely used and has become the standard protocol published by the National Renewable Energy Laboratory (NREL) in 2018 for determining the biopolymer composition in cellulosic wastes [3,4,6–11]. Detailed mechanisms of the reaction have been well described in the chemical literature [12]. The fact that H_2_SO_4_ is a strong acid (pK_a1_ = −3) enables the hydrolysis reaction to complete in a shorter period. Several studies have performed H_2_SO_4_-catalysed reaction for obtaining glucose from WP. A reaction between paper towel and 5% (v/v) H_2_SO_4_ at 135°C for 2 h yielded 30.7 gL^−1^ of glucose, corresponding to *ca* 44% conversion [13]. In another study, 94.4% of glucose conversion was achieved from 300 mg of computer printout under the optimized conditions of 3 mL of 70% (w/w) H_2_SO_4_ at 30°C for 5 h [14]. While H_2_SO_4_ offers advantages for the method, its corrosive nature and the necessity for an additional neutralization step present significant drawbacks, particularly concerning environmental and safety issues.

Enzymatic hydrolysis offers a greener option to the H_2_SO_4_-catalysed reaction [3,4,11]. Utilizing free-cellulase enzyme, the reaction can achieve up to 100% cellulose-to-glucose conversion, generating water as the waste under milder operation conditions [3,4]. The catalytic actions of the enzyme have been extensively reported. Chemically, the presence of amino acid residues provides acidic and basic sites for the hydrolysis of β-1,4-glycosidic linkages [15]. From a biochemical perspective, cellulase enzyme comprises multi-components with different substrate specificities. These multi-components work cooperatively to break down cellulose into glucose monosaccharide. They are endo-glucanase (EG), exo-glucanase (CBH) and β-glucosidase (BG) [3,16,17]. EG initiates the hydrolysis by randomly cleaving internal bonds, disrupting the crystalline structure of cellulose. CBH then targets the non-reducing ends, releasing cellobiose. Finally, cellobiose is further hydrolysed by BG [3,4,17,18]. The success of producing glucose from various types of WP via enzymatic hydrolysis has been widely reported. Hydrolysing office paper using cellulase from *Thermoascus aurantiacu* at 60°C for 24 h released 830 mg mL^−1^ of glucose, corresponding to 83% of conversion [19]. Meanwhile, different types of WP (newspaper, office paper, magazines and cardboard) were hydrolysed employing Celluclast 1.5L supplemented with Novozyme 188 at pH 4.8, 50°C for 72 h, releasing glucose yields of 57% (magazine), 61% (newspaper), 69% (cardboard) and 82% (office paper) [20]. These reported studies show that enzymatic hydrolysis requires a prolonged period to complete, in contrast to the shorter duration required by the H_2_SO_4_-catalysed hydrolysis. This can be explained by the limited accessibility of the enzyme due to the complex and highly crystalline structure of cellulose. Additionally, the fact that enzyme multi-components targeting specific sites along the cellulose chains slows the overall rate of hydrolysis. Despite being time-consuming, enzymatic hydrolysis remains a promising method.

In this study, we compare the experimental procedures, techno-economic evaluation as well as advantages and drawbacks of H_2_SO_4_ (SA)- and free enzyme (FE)-catalysed hydrolysis methods for converting WP (ordinary A4 printing paper) to glucose. The major goal is to assess the overall viability and more importantly, the economic competitiveness of each method, addressing a gap in previous studies that focused solely on reaction optimization and glucose yield. We accomplished this by integrating essential information and associated costs into the Aspen HYSYS, a software designed for estimating chemical production costs [4,8,21]. Note that optimizing reaction conditions for higher glucose yields is not the central objective of this study. We adhered to the NREL standard protocols with minor modifications for both hydrolysis methods [6,22].

## 2. Methodology

### 2.1. Preparation of waste paper

WP (ordinary A4 printing paper, thickness 108 ± 4% µm) was collected from a nearby office, shredded using a mechanical shredder machine, and cut into smaller pieces with dimensions of 0.5 cm (width) × 4 cm (length).

### 2.2. Characterization of waste paper

Surface morphology was examined by a scanning electron microscope (SEM, Hitachi TM-3000 Japan) under the following settings: voltage potential of 5.0 kV, working distance of 5.7–6.9 mm and magnification range from 200× to 1000×. WP was first coated with 2−6 nm of gold (Au) using a sputter coater prior to the analysis. Surface elemental composition and mapping were obtained using an energy-dispersed X-ray (EDX, Oxford Instruments, Ultimax 65) attached to a field emission scanning electron microscope (FESEM, JEOL, JSM IT 800 SHL). Thermal decomposition profile was recorded by a thermogravimetric analyser with high-temperature capability (TGA/HT) DCS HSS2 instrument under the following conditions: heating rate of 15°C min^−1^, N_2_ flow rate of 10 mL min^−1^ and heating temperature ranged from 30°C to 650°C.

### 2.3. Sulfuric acid-hydrolysis of waste paper

**Figure 1.**
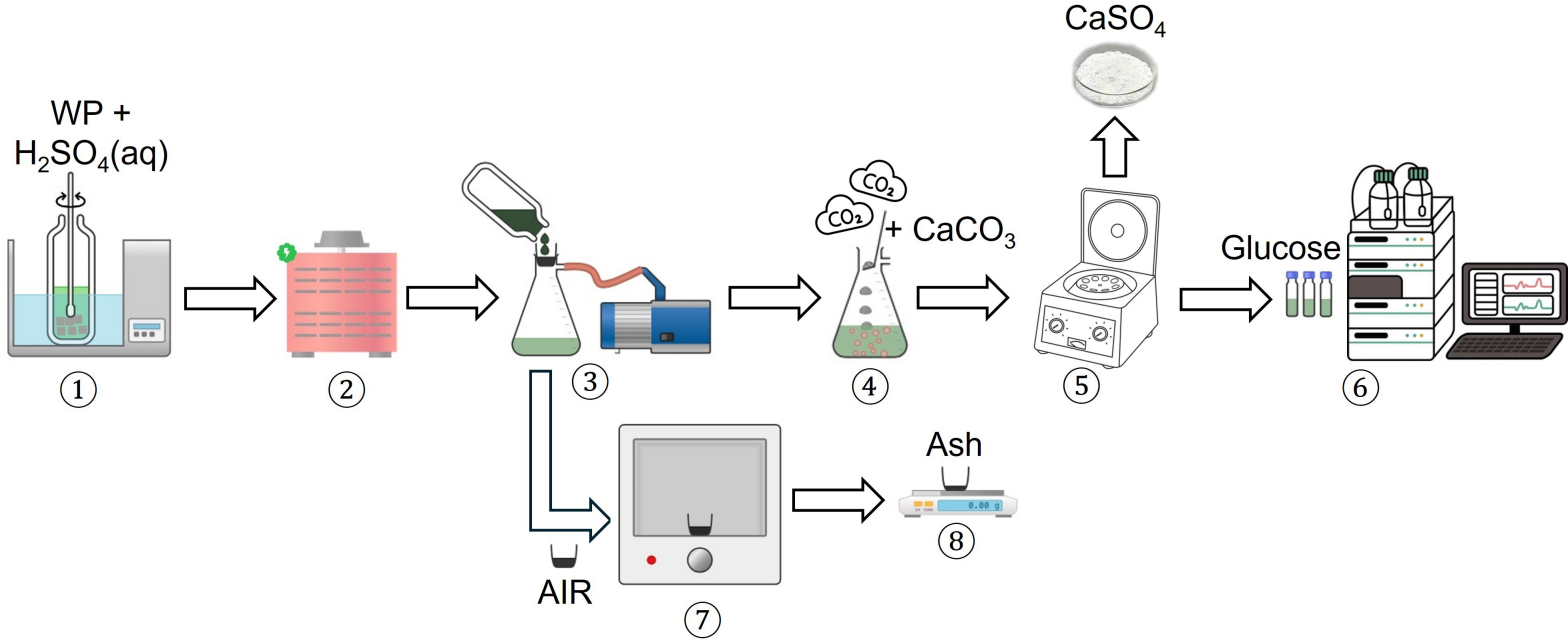
Procedure for SA-hydrolysis adopted from the NREL protocol [6].

The protocol published by NREL was followed [6]. Figure 1 summarizes the procedure. H_2_SO_4_ (72% w/w, 3 mL) was first added to an Ace pressure tube containing WP (300 mg). The tube was incubated in a water bath at 30°C for 1 h, with the mixture stirred using a glass rod every 10 min interval without removing the rod during stirring. Next, distilled water (84 g) was added to the mixture, diluting the concentration of H_2_SO_4_ to 4% w/w. The tube was sealed tightly, inverted several times and autoclaved for 1 h at 121°C and 0.1 mbar. When the autoclave had completed, the mixture was filtered through an ashed crucible under reduced pressure, separating the filtrate from a solid fraction labelled as ‘acid insoluble residue (AIR)’. Calcium carbonate (CaCO_3_) was then added to the filtrate to neutralize H_2_SO_4_, with the base addition continuing until the pH of the resultant mixture reached 5−6. The mixture was later allowed to stand, separating a white calcium sulfate (CaSO_4_) precipitate and a liquid product. Finally, the liquid product was filtered through a 0.2 μm PTFE syringe-filter and later analysed by high-performance liquid chromatography (HPLC). The HPLC analysis was conducted on a Shimadzu Analytical system equipped with an Aminex HPX-97P column (Bio-Rad, 300 × 7.8 mm) and a refractive index (RI) detector using purified water as mobile phase at 0.6 mL min^−1^, with the column temperature of 85°C. The following calibration standards were used: mixed sugars (glucose, xylose, mannose, arabinose and galactose) with concentrations of 0.1, 1, 2 and 4 mg mL^−1^ and only glucose at 8 mg mL^−1^. HPLC graphs can be found in electronic supplementary material, figure S1. Meanwhile, the recovered AIR in the ashed crucible placed in a muffle furnace was heated at 570°C for 3 h. The resultant ash was weighed, and its mass was recorded.

### 2.4. Free cellulase enzyme-hydrolysis of waste paper

**Figure 2.**
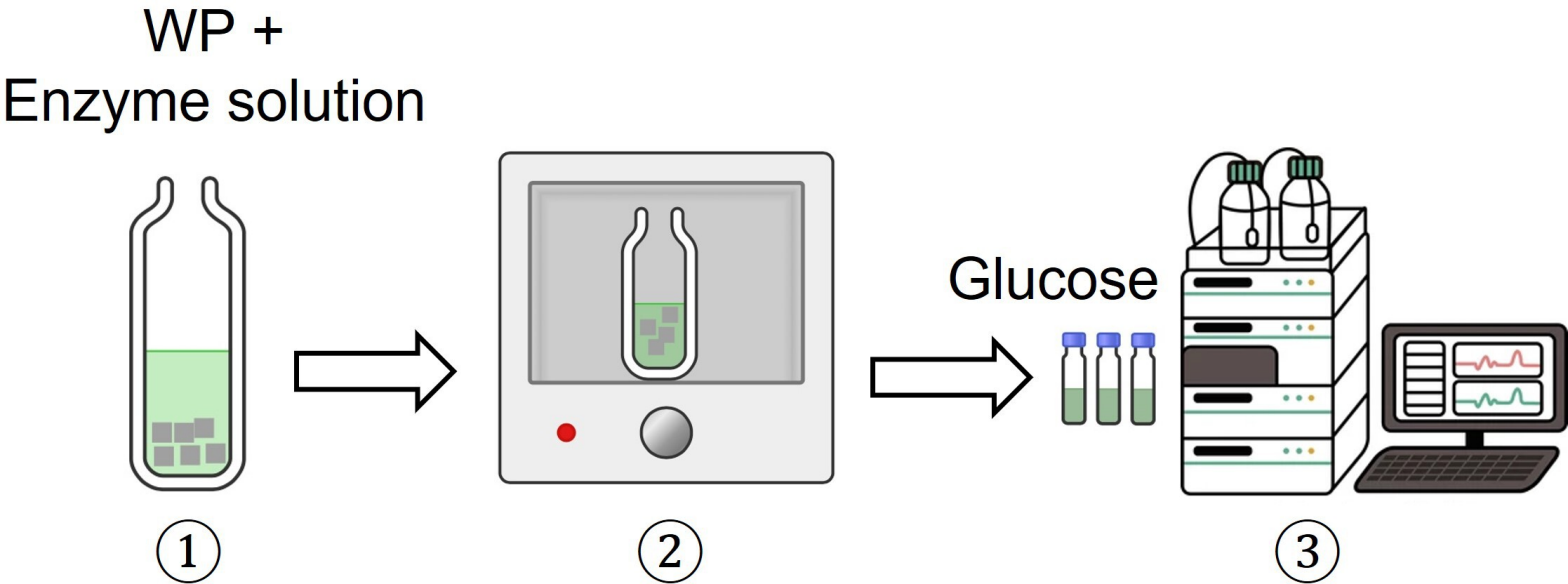
Procedure for FE-hydrolysis.

A published protocol was referred with minor modifications [7,9,11]. The following materials were added to a 50 mL falcon tube: WP (100 mg), 0.1 M citrate buffer solution (pH 4.7, 5 mL), tetracycline (80 μL) and cellulase enzyme (*Trichoderma reesei*, greater than or requal to 700 units g^−1^, 120 μL). Distilled water was then transferred into the tube to increase the total volume to 10 mL. Next, the tube was incubated in an incubator at 50°C and shaken at 150 r.p.m. for 72 h. When the incubation period had elapsed, the sample was immediately cooled in an ice bath, filtered through a NTFE syringe and analysed on a Shimadzu Analytical system equipped with an Aminex HPX-97P column (Bio-Rad, 300 × 7.8 mm) and an RI detector using purified water as mobile phase at 0.6 mL min^−1^, with the column temperature of 85°C (graph can be found in electronic supplementary material, figure S2). The following calibration standards were used: mixed sugars (glucose, xylose, mannose, arabinose and galactose) with concentrations of 0.1, 1, 2 and 4 mg mL^−1^ and only glucose at 8 mg mL^−1^ (see electronic supplementary material, figure S1(b)-(f)). The undigested residue was collected, dried in an oven at 150°C overnight and later weighed. Figure 2 shows the overall procedure.

### 2.5. Process modelling

Aspen HYSYS v. 12 software was used to compute the mass and energy balances for both SA-hydrolysis and FE-hydrolysis. Sizing parameters for the reactor and unit operation were derived either directly or indirectly from the Aspen HYSYS simulation, while sizing parameters for the heat exchanger were estimated through the Aspen energy analyser. Reactors were represented as jacket-agitated vessels based on their volume, which was estimated from the volumetric flow rate at the reactor outlet, the reaction temperature and the residence time of the reactor. The effects of thermodynamics and operating conditions on glucose yield were disregarded. Instead, the glucose yield and operating parameters were kept constant to maintain consistency in the techno-economic analysis. Table 1 lists the feedstock quantities and operating conditions employed in the process simulation, assuming that each method can process 60 kg h^−1^ of WP. The calculations for the feedstock quantities using the laboratory scaling factor are described in electronic supplementary material, table S1. WP was assumed to contain solely cellulose.

**Table 1.** Feedstock quantities and operating conditions of SA-hydrolysis and FE-hydrolysis methods.

	SA-hydrolysis	FE-hydrolysis
waste-paper (WP)	60 kg h^−1^	60 kg h^−1^
sulfuric acid (72 wt%)	978 kg h^−1^	—
water	16 800 kg h^−1^	2940 L h^−1^
calcium carbonate	997.94 kg h^−1^	—
cellulase	—	12 L h^−1^
citrate buffer	—	3000 L h^−1^
tetracycline	—	48 L h^-1^
temperature (°C)	121	50
pressure (atm)	0.001	1
total inlet molar flow (kg mole h^−1^)	952.91	117.97

Hypothetical components used in the simulation, including their specific properties and data sources, are given in table 2. For unlisted components, their properties are available in the HYSYS database.

**Table 2.** Hypothetical components and their properties.

hypothetical component	specified properties				references
	molecular weight (g mol^−1^)	boiling temperature (°C)	density (kg m^−3^)	critical temperature (°C)	
cellulose	162.1	—	1500	—	Humbird *et al.* [23]
citric acid monohydrate	210.1	86.99	21.01	2142	PubChem CID: 2230
tetracycline	444.4	457.2	1700	743.3	PubChem CID: 546 75776
cellulase enzyme	504.4	55.00	1800	282.7	PubChem CID: 4 40950

### 2.6. Techno-economic analysis

The following costs were predicted by the Aspen simulation in US dollars with related assumptions described in electronic supplementary material, table S2: total capital, total operating, total raw material and total utilities. Each cost was detailed with specific components that contributed to the overall expense. Table 3 lists the prices of chemical feedstocks as quoted by Sigma-Aldrich at the time of writing.

**Table 3.** Prices of chemical feedstocks used in the simulation.

materials	price/unit
sulfuric acid	$99.6 L^−1^
calcium carbonate	$112 kg^−1^
citrate buffer solution	$1.36 L^−1^
cellulase enzyme	$2700 L^−1^
tetracycline	$26.6 g^−1^
glucose	$199 kg^−1^

## 3. Results and discussion

### 3.1. Characterization of waste paper

**Figure 3.**
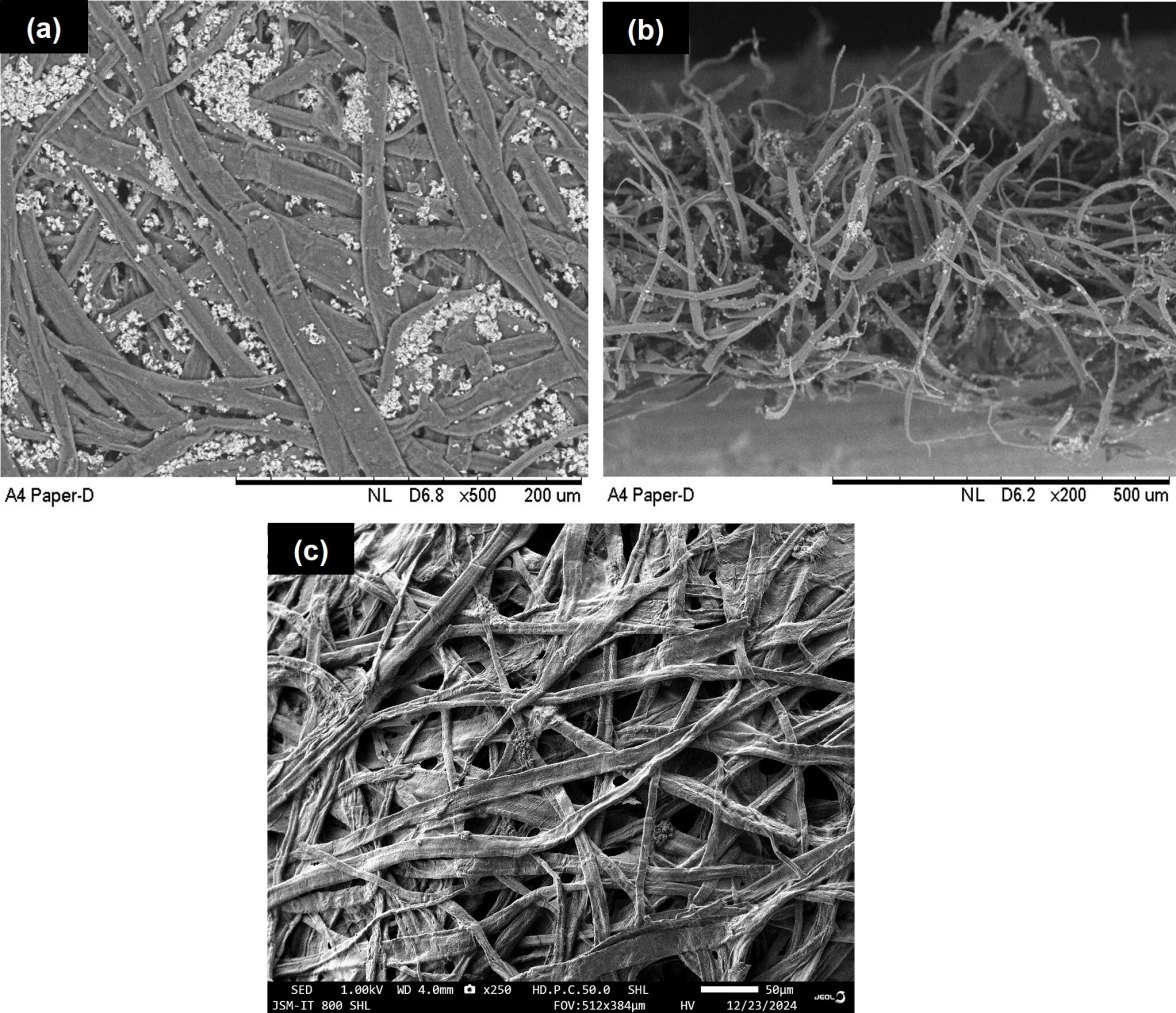
SEM images of (a) WP from top view, (b) WP from cross-sectional and (c) undigested residue recovered after WP was subjected to FE-hydrolysis at 50°C for 72 h.

Figure 3(a) shows the top-view morphology of WP, exhibiting networks of elongated fibres that are cross-linked. In addition, small agglomerated particles appear distributed across some areas and within the fibre matrix. Interestingly, the cross-sectional view in figure 3(b) shows that the fibres are loosely arranged, with some surfaces covered by agglomerated particles. In both images (figure 3(a,b)), the agglomerated particles have a lighter contrast against the darker fibre surfaces. This strongly indicates that these particles were composed of chemical elements with higher atomic numbers, which scatter more electrons during the SEM analysis.

**Figure 4.**
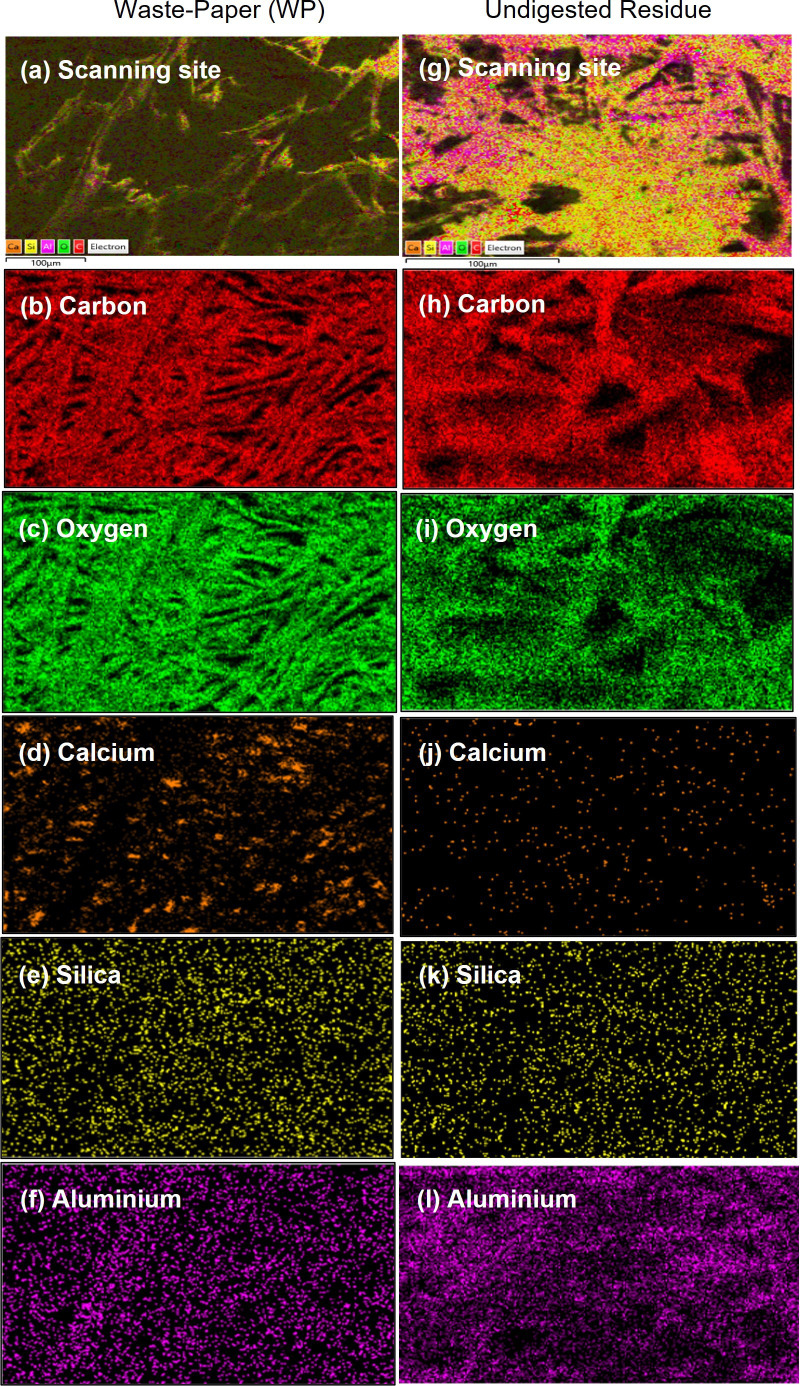
Surface-elemental distribution images recorded by EDX for WP: (a) scanning area, (b) carbon, (c) oxygen, (d) calcium, (e) silica and (f) aluminium. Images for undigested residue recovered after WP was subjected to FE-hydrolysis at 50°C for 72 h: (g) scanning area, (h) carbon, (i) oxygen, (j) calcium, (k) silica and (l) aluminium.

WP was then analysed by EDX. As depicted in figure 4(d–f), the fibre surfaces are extensively covered by Ca, Si and Al elements, with their compositions presented in table 4 (micrographs can be found in electronic supplementary material, figure S3). Note that these values are semi-quantitative and thus not highly accurate. This is because EDX provides surface composition, not bulk composition. What is more important is that the detection of these elements by EDX appears to confirm that the agglomerated particles visible in SEM images (figure 3(a,b)) are composed of Ca, Si and Al. They are chemical elements of inorganic fillers added during the paper production. Common fillers include calcium carbonate (CaCO_3_) and kaolin (Al_2_Si_2_O_5_(OH)_4_), which are distributed between the spaces of fibres [24].

**Table 4.** Surface elemental compositions of WP and undigested residue analysed by EDX. Error values (±) are the s.d. of data recorded at four different sites.

elements	weight %		atomic %	
	WP	undigested residue^a^	WP	undigested residue
carbon	48.9 ± 0.14	60.4 ± 1.19	56.6 ± 0.133	67.6 ± 1.11
oxygen	49.5 ± 0.04	37.3 ± 1.27	42.9 ± 0.067	31.4 ± 1.76
calcium	1.29 ± 0.11	0.035 ± 0.008	ND	ND^b^
silica	0.035 ± 0.005	0.028 ± 0.007	0.1 ± 0.001	ND
aluminium	0.163 ± 0.01	0.035 ± 0.08	0.467 ± 0.067	1.05 ± 0.05
C/O ratio	0.996	1.62	1.32	2.15

^a^ Undigested residue refers to the undigested material recovered following the completion of FE-hydrolysis of WP at 50°C for 72 h.

^b^ ND denotes no reading detected by EDX analysis.

**Figure 5.**
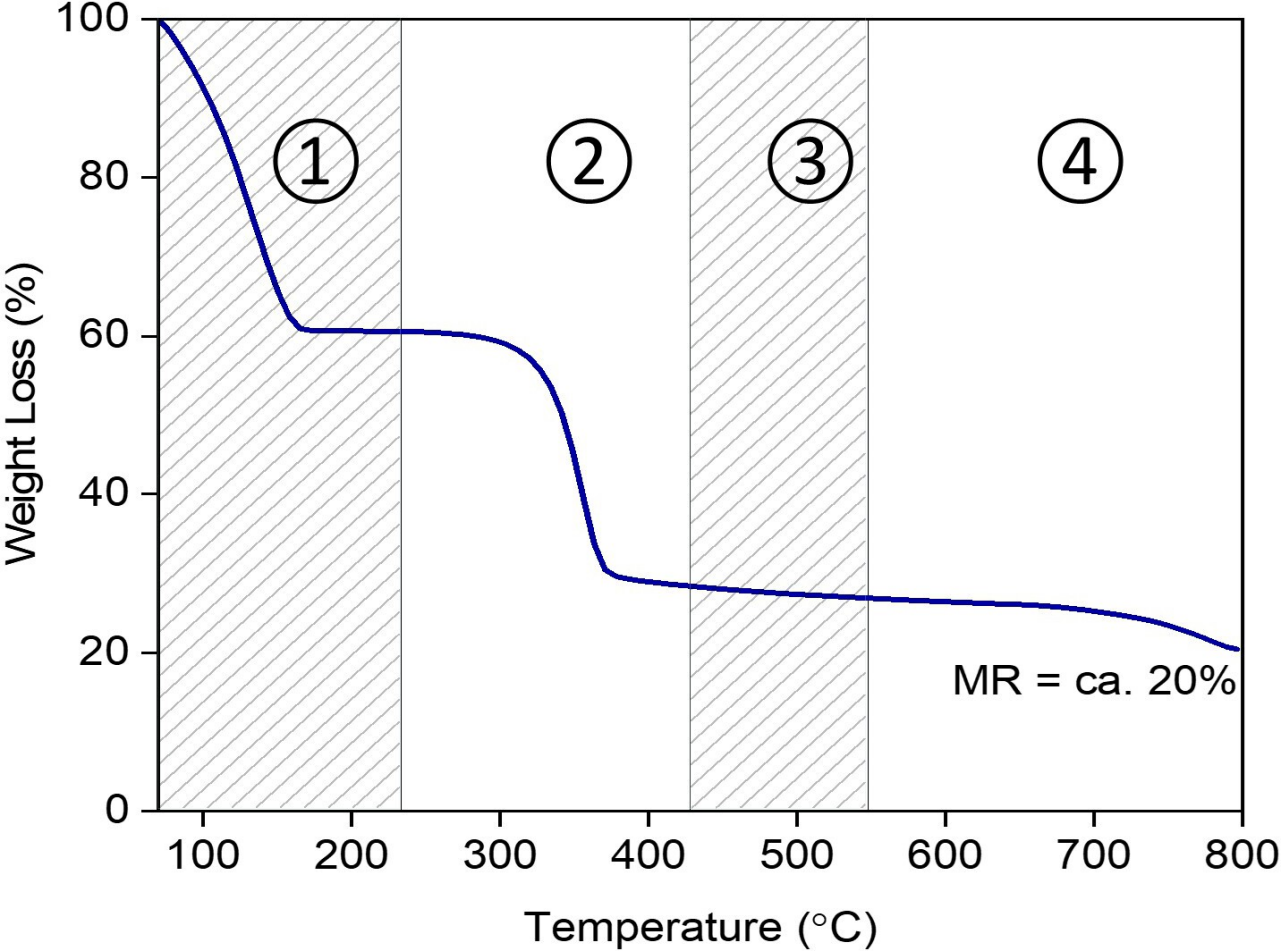
Decomposition profile of WP by TGA.

Thermal decomposition of WP was evaluated by TGA. As shown in figure 5, the decomposition is divided into four stages, as proposed previously [25]. *Stage 1*, with *ca* 40% of weight loss occurring up to *ca* 230°C, probably reflects the removal of unbound moisture, as well as the decomposition of hemicellulose and lower molecular-weight compounds. *Stage 2*, from 230°C to 430°C, can be attributed to the decomposition of cellulose and hemicellulose fractions, resulting in *ca* 38% of weight loss. The decomposition continues in *Stage 3* up to 548°C, probably indicating the decomposition of cellulose and higher molecular weight compounds. *Stage 4*, occurring above 548°C, can be assigned to the decomposition of lignin, which requires much higher temperature than other constituents due to its polyphenol aromatic polymer structure with a higher order of cross-linked connections [25]. At 800°C, *ca* 20% of mass residue (MR) is recorded, representing char residue. Char is the major product of lignin pyrolysis [26]. It was also believed that the presence of Ca, Si and Al in WP (as shown in figure 4(d–f) and table 4) also contributed to the formation of char residue, as these elements in the form of oxides are thermally stable at very high temperatures.

### 3.2. Hydrolysis of waste paper

**Figure 6.**
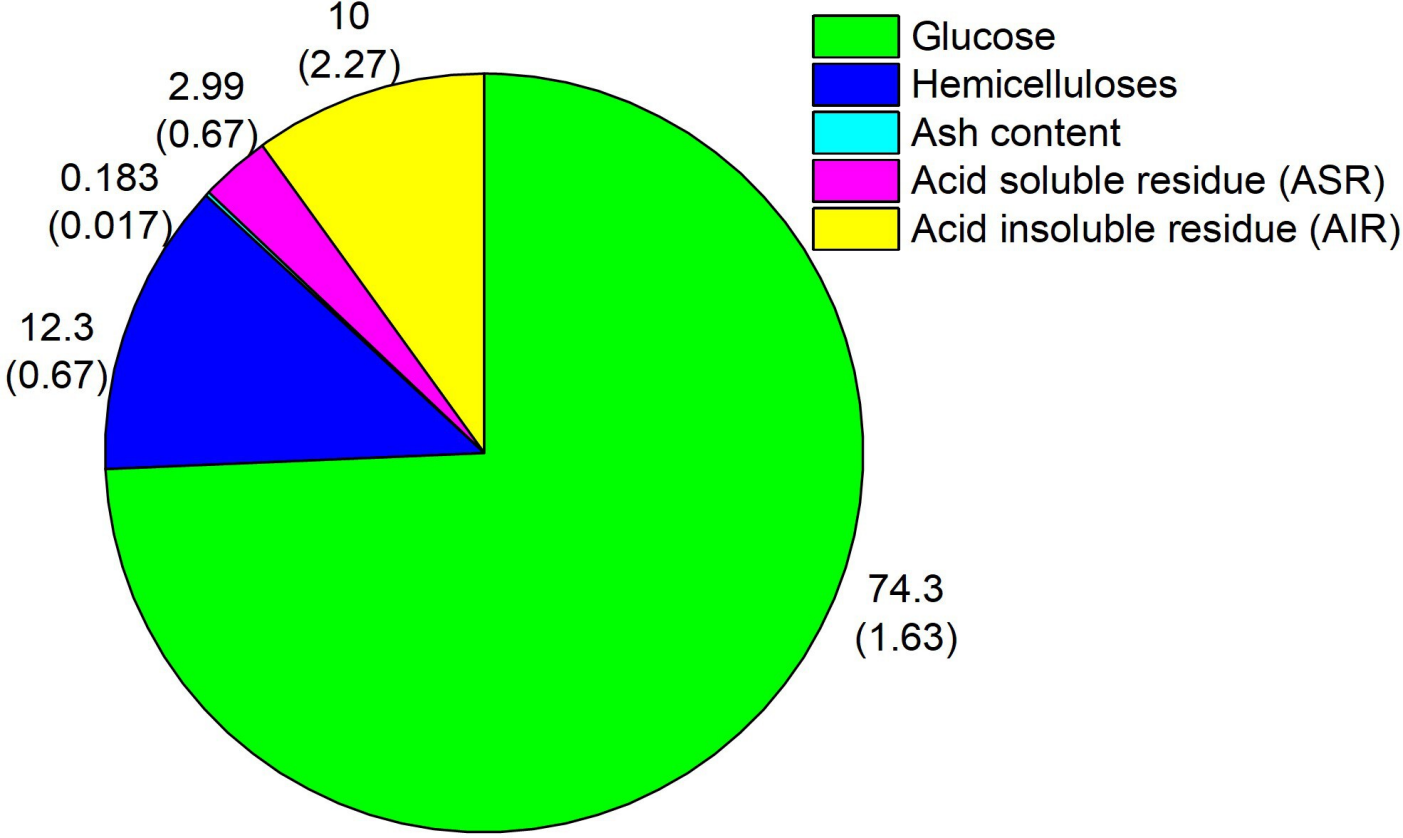
Outcomes of SA-Hydrolysis.

Figure 6 depicts the results of SA-hydrolysis. As expected, cellulose appears as the major constituent of WP. SA-hydrolysis method is an established protocol for determining the biopolymer compositions in cellulosic wastes, where glucose and hemicellulose sugar measurements directly reflect cellulose and hemicellulose contents [6–11,27–29].

The analysis also recorded a small fraction of AIR (10%), recovered after the reduced-pressure filtration (figure 1, Step 3). The AIR is very likely a predominant lignin fraction, which is highly insoluble in H_2_SO_4_. This, in fact, is a typical outcome of SA-hydrolysis of cellulosic wastes, like biomass [8–11,27,29,30]. The insolubility of lignin in H_2_SO_4_ is mainly due to aromatic and hydrophobic characters of the polymer, as thoroughly explained in the chemical literature [31–33]. The presence of lignin in AIR is further evidenced by *ca* 98% weight loss after AIR was subjected to open combustion in the muffle furnace (figure 1, Step 7), leaving 0.18% of ash. Meanwhile, the presence of ash strongly suggests that the AIR also contained small quantities of minerals, particularly Ca, Al and Si as detected by EDX analysis (figure 4). These minerals are highly stable when heated at high temperatures. Notably, there is *ca* 3% of acid soluble residue (ASR), referring to compounds that were neither detected by HPLC nor recovered as a solid fraction after under-pressure filtration (figure 1, Step 3). We strongly believed that ASR might have contained low-molecular-weight lignin fragments that were not recoverable during the reduced-pressure filtration (figure 1, Step 3) and subsequently carried away in the glucose-H_2_SO_4_ mixture.

**Figure 7.**
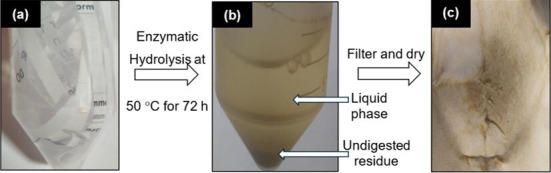
Hydrolysis of WP via FE-hydrolysis method at 50°C for 72 h: (a) pristine WP, (b) slurry produced and (c) undigested residue.

Hydrolysis of WP via FE-hydrolysis resulted in a slurry with a liquid phase, containing glucose (75.9 ± 1.48%) and hemicellulose sugars (1.36 ± 0.036%), along with undigested residue (33.2%) (figure 7). Note that the glucose yield (75.9 ± 1.48%) exceeds *ca* 2% of glucose content determined by SA-hydrolysis (figure 6). This small increase could be due to the presence of traces of glucose introduced during the enzyme preparation from *T. reesei*, as previously reported [34–36]. Above all, the results deduced that the entire cellulose content in WP was hydrolysed to glucose via FE-Hydrolysis. Meanwhile, a small fraction of hemicellulose was hydrolysed, which is unexpected given the fact that cellulase’s specificity is for cellulose substrate. A likely reason is due to the presence of small amounts of hemicellulase in the enzyme solution used. In general, *T. reesei* also produces hemicellulase in lower quantities, including xylanases [37–39]. A large amount of undigested residue (33%) was recovered following the completion of FE-hydrolysis (figure 7(c)), most likely containing hemicellulose, lignin and inorganic fillers. However, the value is *ca* 25% higher than anticipated, probably caused by physically bound water molecules and/or residual enzyme that was retained on the undigested residue.

We also characterized the undigested residue (figure 7(c)) by SEM and EDX and compared the outcomes with those recorded for WP. Compared with WP in figure 3(a), the fibres appear more individual, with less cross-linking and a noticeable absence of agglomerated particles (figure 3(c)). In addition, their structural remains fibrillated with no alterations, such as swelling. This is expected as cellulase enzyme selectively hydrolyses cellulose, leaving hemicellulose and lignin intact. However, it is difficult to visually differentiate whether the fibres depicted in figure 3(c) are hemicellulose and/or lignin under a microscope. This is because these polymers are composed of the same characteristic elements: C, H and O. As a result, clear contrast is difficult to obtain. Moreover, there is no distinct morphological identity between cellulose, hemicellulose and lignin. The surface composition in table 4 reveals that the C/O ratio (weight and atomic %) for the undigested residue is much higher than WP, indicating enhanced surface exposure of C and O to the electron beam during EDX analysis. This is attributed to the decreased presence of inorganic fillers, as evidenced by reduced elemental distributions and quantities of Ca, Si and Al on the surface of the undigested residue (figure 4(j–l) and table 4). These fillers might have leached out from the fibre matrix during 72 h of FE-hydrolysis.

Relating FE-hydrolysis outcomes with characterization results (figures 3–5) unveils interesting findings. Firstly, the complete conversion of cellulose in WP to glucose strongly reflects increased enzyme binding to cellulose. This is probably a result of the more exposed and loosely packed cellulose microfibrils, as exhibited by SEM images in figure 3(a,b). Technically, the more exposed the microfibrils, the higher the enzyme–cellulose binding, leading to increased glucose yield. Additionally, the complete cellulose-to-glucose conversion strongly indicates hemicellulose, lignin and inorganic fillers in WP (figure 4(d–f), figure 6 and table 4) did not impact enzyme–cellulose binding, suggesting that they were not as strongly bound to cellulose in WP. The weak attachment of the hemicellulose-lignin fraction to cellulose may be linked to the nature of the pulping process in paper production, which breaks many covalent bonds that would otherwise tightly bind the hemicellulose-lignin fractions to cellulose. Regarding inorganic fillers, the additives generally occupy tiny spaces within the cellulose sheets via physical insertion [40], and we believed they do not hinder enzyme–cellulose binding. Further examination is currently underway to examine these aspects in greater detail.

### 3.3. Process design

#### 3.3.1. Sulfuric acid-hydrolysis

**Figure 8.**
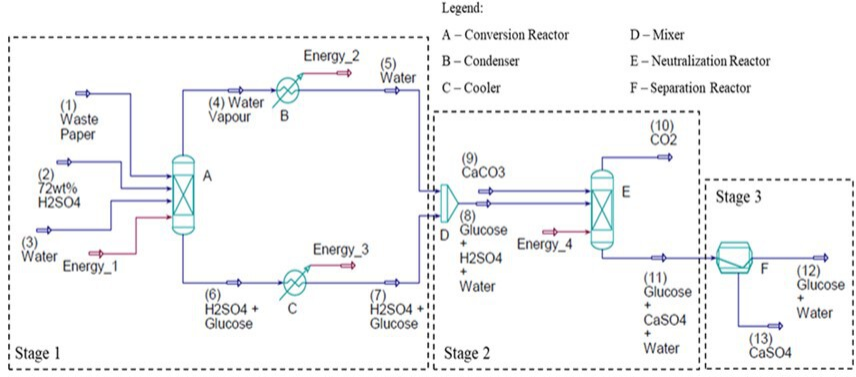
Process design of SA-hydrolysis simulated by the Aspen HYSYS software. The simulation assumed complete hydrolysis conversion with 100% glucose yield.

The simulated design is illustrated in figure 8 and is divided into three stages. In *Stage 1— Digestion and Hydrolysis*, WP is first added to an agitated reactor (**A**), followed by the addition of 72 wt% H_2_SO_4_. The mixture undergoes pre-hydrolysis reaction, where it is heated at 30°C under continuous stirring for 1 h. Upon completion, water is added to the reactor, reducing the concentration of H_2_SO_4_ in the mixture to 6 wt%. Subsequently, the reactor is further heated at 121°C with continuous stirring under a pressure of 0.1 bar. After the heating stage process is completed, a condenser (**B**) cools water vapour emitted from the reactor’s top output (**A**) to 25°C, and the resultant condensed water is later fed into a mixer (**D**). Concurrently, a solution, containing the product glucose and H_2_SO_4_, is released from the reactor’s bottom outlet, cooled to room temperature by a condenser (**C),** and then fed into the mixer (**D**).

The mixture (containing glucose, H_2_SO_4_ and water) in the mixer (**D**) undergoes *Stage 2— Neutralization*, in which it is charged into a second reactor (**E**). CaCO_3_ is added into the reactor to neutralize H_2_SO_4_, forming insoluble solid CaSO_4_ suspended in glucose solution, as well as emitting CO_2_. Finally, the glucose solution and insoluble CaSO_4_ enter *Stage 3—Separation*, where they are separated in a rotary vacuum filter (**F**).

#### 3.3.2. Free cellulase enzyme-hydrolysis

**Figure 9.**
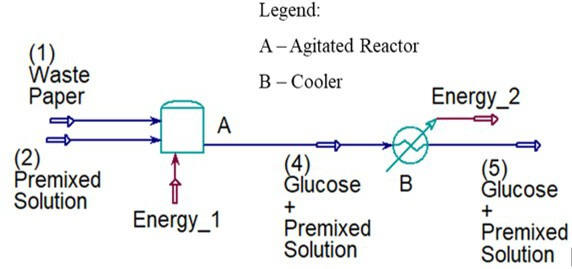
Process design of FE-hydrolysis simulated by the Aspen HYSYS Software. The simulation assumed complete hydrolysis conversion with 100% glucose yield.

As depicted in figure 9, the procedure begins by loading WP into an agitated reactor (**A**), followed by adding a premixed solution containing the following chemicals: cellulase enzyme, tetracycline, citrate buffer solution and water. Subsequently, the reactor is heated at 50°C with continuous agitation under atmospheric pressure for 72 h. When the period of heating process elapses, the resultant mixture is immediately passed through a cooler (**B),** deactivating the enzyme activity.

### 3.4. Process input and output

Table 5 shows the full composition of all feedstocks and end-products. The simulation assumed processing 60 kg h^−1^ of WP, with the operational capacity at 8766 h yr^−1^ to ensure year-round output. In terms of glucose production, FE-hydrolysis yields 17 times less glucose compared with SA-hydrolysis. Technically, SA-hydrolysis with its shorter hydrolysis time enables more processing cycles per year and consequently higher glucose output, as shown in table 5. Despite the higher glucose output, SA-hydrolysis consumes H_2_SO_4_ and CaCO_3_ in quantities 12 times greater than the glucose produced and generates CaSO_4_ at 21 times the glucose output with a large quantity of CO_2_. In contrast, FE-hydrolysis method produces zero by-products despite producing a far lower glucose output.

**Table 5.** Glucose production for SA-hydrolysis and FE-hydrolysis.

input	^a^flow rate (kg yr^−1^)	
	SA-hydrolysis	FE-hydrolysis
complete reaction period per cycle (h)	2	72
waste paper (WP)	262 980.00	7305.00
sulfuric acid	3 480 102.00	—
calcium carbonate	3 554 963.64	—
water	73 634 400.00	357 945.00
citrate buffer	—	365 250.00
tetracycline	—	5844.00
cellulase enzyme	—	1461.00
output		
^b^glucose	288 464.05	16 233.55
calcium sulfate	6 110 048.46	—
carbon dioxide	177 522.62	—
water	73 292 976.32	349 016.63
citrate buffer	—	365 250.00
tetracycline	—	5844.00
cellulase enzyme	—	1461.00

^a^ Mass flow of each material stream.

^b^ Glucose quantity produced during dynamic phase of the simulation. The complete reaction period is 2 h/cycle for SA-hydrolysis and 72 h/cycle for FE-hydrolysis.

### 3.5. Economic viability projection

Table 6 compares total process costs of SA-hydrolysis and FE-hydrolysis projected by the Aspen Process Economic Analyser (APEA) v. 12. The details of the assumptions, equations and estimations utilized in this assessment are provided in the electronic supplementary material, table S2.

**Table 6.** Projected process costing for the hydrolysis of WP to glucose.

cost	SA-hydrolysis	FE-hydrolysis
total capital ($ million)	4.53	3.23
total operating ($ million yr^−1^)	1.47	0.85
total raw material ($ million yr^−1^)	658.9	3.86
total utilities ($ million yr^−1^)	0.14	0.0005
total glucose sales ($ million yr^−1^)	57.4	3.23
profit ($ million yr^−1^)^a^	−603.1	−1.48

^a^ Profit is the total glucose sales minus with the sum of the following total costs: operating, raw materials and utilities.

#### 3.5.1. Capital cost

**Figure 10.**
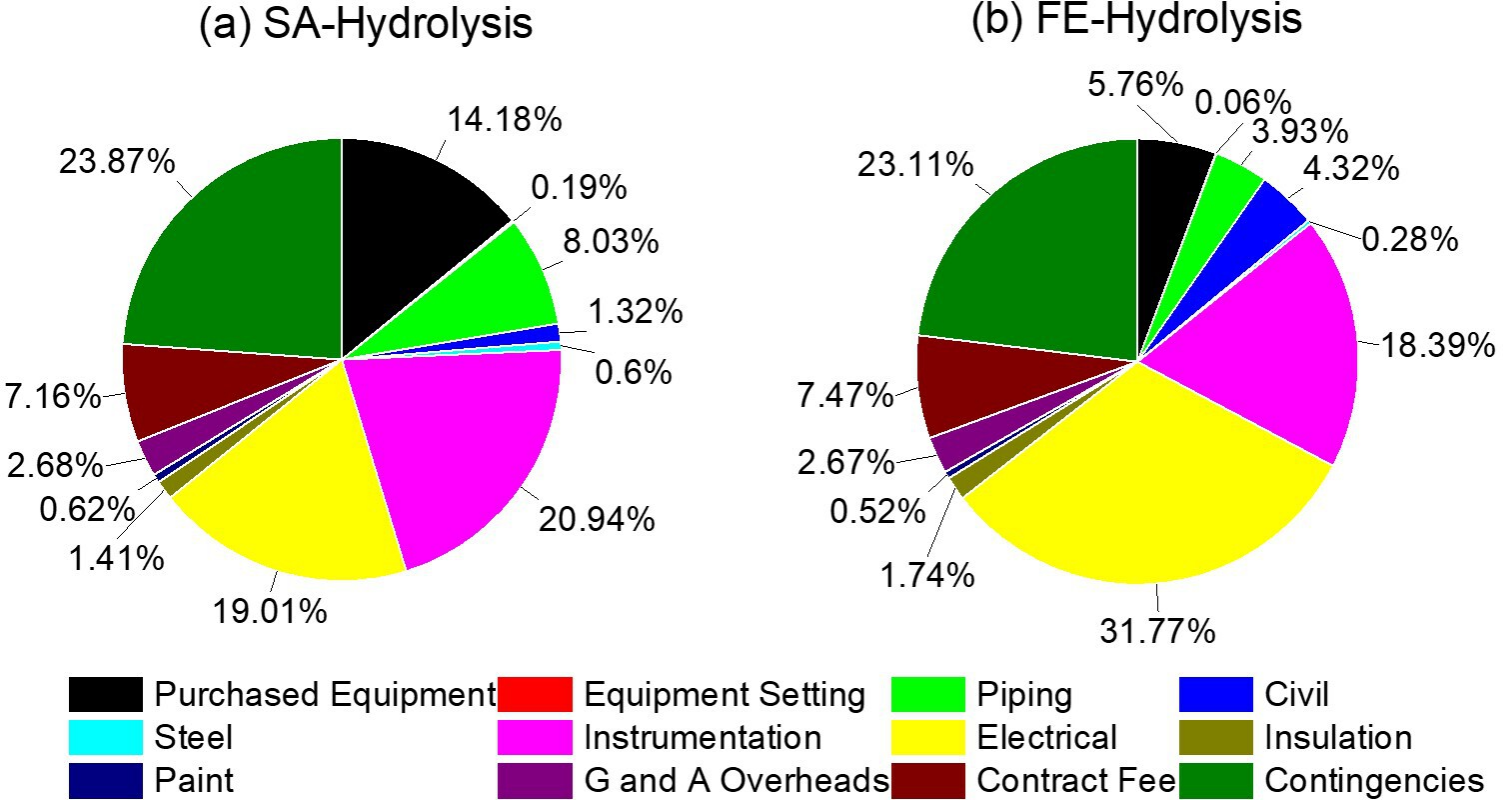
Cost components and their individual contributions to the total capital cost for (a) SA-hydrolysis and (b) FE-hydrolysis.

The cost describes all the out-pocket expenses, encompassing all of the fixed, one-time costs associated with the following elements: purchasing buildings, machinery, equipment, infrastructure, engineering, design and others directly related to the creation of the asset. Figure 10 summarizes cost components and their individual contributions to the total capital cost for both hydrolysis methods (table 6, entry 1). Relative to FE-hydrolysis, SA-hydrolysis costs *ca* two times more for piping and *ca* 13% more for instrumentation. The higher costs reflect the complexity of the process involving multiple steps, as illustrated in figure 8. Expectedly, the electrical cost for FE-hydrolysis is *ca* 1.6 times higher than for SA-hydrolysis, primarily because FE-hydrolysis requires 72 h to complete, while SA-hydrolysis takes only 2 h.

#### 3.5.2. Total operating cost

**Figure 11.**
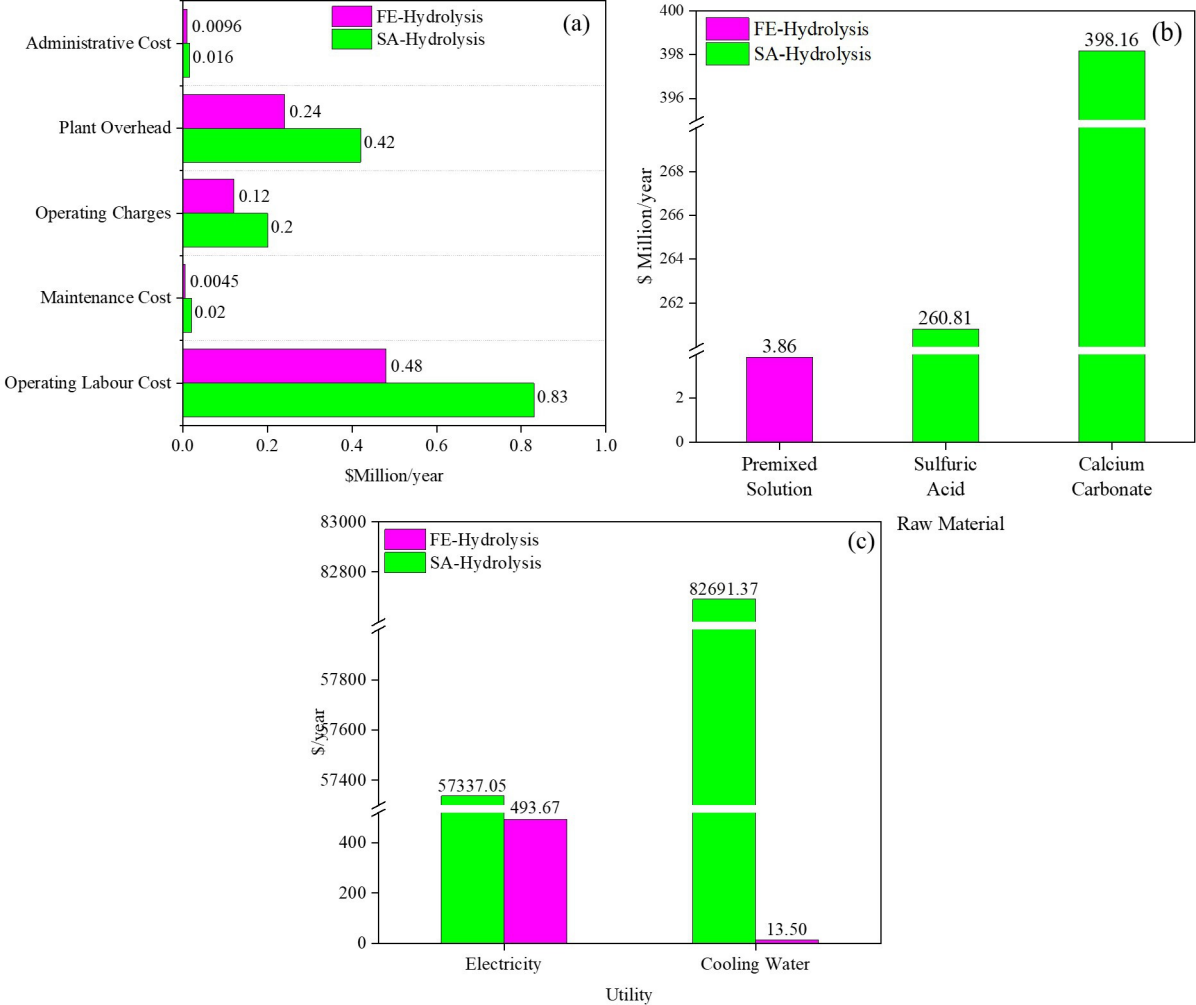
Operating cost elements for (a) total operating cost, (b) total raw material cost and (c) total utility cost.

Figure 11(a) summarizes the expenses for daily product manufacturing and their contributions to the total operating cost (table 6, entry 2). Overall, all expenses related to SA-hydrolysis are higher, with operating labour cost being *ca* 73% greater than that for FE-hydrolysis.

#### 3.5.3. Total raw material cost

The cost covers the purchase of raw materials for the process but excludes shipping, handling, or storage fees. Figure 11(b) summarizes the outcomes, showing that for SA-hydrolysis, the purchase of H_2_SO_4_ accounts for 39.5% and CaCO_3_ for 60.4% of the total raw material cost of $658.97 million per year (table 6, entry 3). In contrast, the cost of the premixed solution (containing citrate buffer solution, tetracycline, cellulase enzyme and water) for FE-hydrolysis is significantly lower, about *ca* 170 times less than the raw materials for SA-hydrolysis.

#### 3.5.4. Total utility cost

The cost covers the consumption of utilities during the production. Overall, the total utility cost for SA-hydrolysis is far higher than for FE-hydrolysis (table 6, entry 4), as shown in figure 11(c).

#### 3.5.5. Profit

Although both methods yield negative profit margin (table 6, entry 6), FE-hydrolysis exhibited a far less negative outcome. This indicates that FE-hydrolysis has the potential to become more economically favourable, with further process optimization and modification.

### 3.6. Advantages and disadvantages of hydrolysis methods

#### 3.6.1. Sulfuric acid-hydrolysis

H_2_SO_4_ is used in the hydrolysis of cellulose to glucose because it is a strong acid. When mixed with water, the acid generates a high concentration of hydronium ions ([H_3_O]^+^), which are a strong Brønsted acid catalyst. The availability of more [H_3_O]^+^ ions leads to protonation of more β−1,4-glycosidic bonds, accelerating the hydrolysis of cellulose. This explains the 2 h total time required to complete the hydrolysis reaction, as specified in the NREL protocol [6]. The catalytic actions of [H_3_O]^+^ ions in hydrolysing β-glycosidic bonds in cellulose are well-documented in the chemical literature. Aside from being a highly efficient catalyst, H_2_SO_4_ is inexpensive and readily available in larger quantities.

However, the use of H_2_SO_4_ is not eco-friendly due to its highly corrosiveness. More importantly, the acid and glucose are highly miscible; thus, it is very challenging to isolate these chemicals individually via either physical or chemical separation means. Prolonged exposure of glucose to H_2_SO_4_, particularly at elevated temperatures, can result in its degradation to yield 5-hydroxymethylfurfural (HMF) via dehydration catalysed by the acid. To address this, CaCO_3_ is added to glucose-H_2_SO_4_ mixture to neutralize the acid, resulting in the generation of CO_2_ and CaSO_4_ solid as by-products. The reaction is irreversible, and the quantity of the by-products generated directly dependent on the amount of H_2_SO_4_ used. This means that the regeneration of H_2_SO_4_ for reuse as a catalyst is practically challenging. The release of CO_2_ is detrimental to the environment. Meanwhile, the production of CaSO_4_ presents several challenges, including the need for proper waste management to avoid soil and water contamination. Moreover, the reuse potential of CaSO_4_ is still limited, leading to accumulation as waste.

#### 3.6.2. Free cellulase enzyme-hydrolysis

Cellulase enzyme offers the benefit of being eco-friendly, specifically breaking down cellulose and achieving nearly 100% glucose yield. Unlike SA-hydrolysis, which generates CO_2_ and CaSO_4_, FE-hydrolysis results in water as the waste, making a much safer chemical process. Although cellulase enzyme has COOH acidic sites, they are insufficiently acidic to facilitate the dehydration of glucose to 5-HMF even at elevated temperatures, unlike the strong acid H_2_SO_4_.

A major disadvantage of using cellulase enzyme is its high cost and limited availability. The high cost makes it economically challenging for large-scale applications. On the other hand, the high miscibility of glucose and enzyme molecules in water complicates their individual separation either through physical or chemical means, leading to difficulties in re-using the enzyme and recycling water back to the process. This negatively impacts the overall process economics.

Another significant drawback is the time-consuming nature of the complete hydrolysis process. Several factors contribute to this extended duration, including the weak acidic nature of COOH, resulting in the generation of fewer [H_3_O]^+^ ions, which are essential for catalysing the hydrolysis reaction. Additionally, cellulase enzymes, which exhibit both endo- and exo-activity, are highly specific and work synergistically by targeting specific regions of the cellulose structure. As a result, the overall hydrolysis kinetics is slower. All these factors collectively contribute to the lengthy duration of the hydrolysis process, limiting FE-hydrolysis’ efficiency and practicality for large-scale application.

## 4. Conclusion

In summary, surface characterizations of WP by SEM and EDX showed that fibres were loosely cross-linked, with inorganic filler elements (Ca, Si and Al) occupying tiny spaces between fibrous sheets. Thermal decomposition analysis by TGA indicated the presence of cellulose, hemicellulose and lignin, while the mass residue corroborated the presence of minerals in WP. Although SA-hydrolysis achieved complete cellulose-to-glucose conversion in a shorter period, the use of corrosive H_2_SO_4_ and the generation of CO_2_ and CaSO_4_ by-products significantly contributed to a much higher total process costing, as projected by the Aspen HYSYS. The raw material cost alone amounted to $658.9 million per year, contributing to the substantial profit loss of $603.1 million per year. As a result, the process can be considered economically uncompetitive. In contrast, FE-hydrolysis also achieved complete cellulose-to-glucose conversion. The projected raw material cost was $3.86 million per year, *ca* 170 times lower than SA-hydrolysis. This in turn resulted in a profit loss of $1.48 million per year, which is *ca* 400 times lower than SA-hydrolysis. Despite this, the extended reaction period required to achieve complete conversion and the difficulty in separating the cellulase enzyme from the glucose solution remain two major drawbacks. Overall, FE-hydrolysis holds significant potential for large-scale implementation over SA-hydrolysis, provided significant process improvements and optimizations are made to reduce reaction period and enhance efficiency further.

## Data Availability

The datasets supporting this article have been uploaded as part of the electronic supplementary material [41].
